# PHYSICAL ACTIVITY PREFERENCES OF OLDER ADULT MEDICAID HOME AND COMMUNITY-BASED SERVICES WAIVER CLIENTS

**DOI:** 10.1093/geroni/igz038.1851

**Published:** 2019-11-08

**Authors:** Victoria S Davila, Lauren Pietzak, Danielle Rossi, Karen Phipps, Margaret Danilovich

**Affiliations:** Northwestern University, Chicago, Illinois, United States

## Abstract

Despite the known effectiveness, physical activity (PA) is not currently offered to older adult clients receiving Medicaid Home and Community Based Services (HCBS). To optimize PA implementation within Medicaid HCBS, understanding client preferences for PA programming is needed. Thus, the objective of this exploratory qualitative study was to identify the PA preferences of HCBS clients including mode, duration, implementation strategy, and frequency, as well as barriers and motivators to PA. We recruited participants from the Illinois’ Department on Aging Community Care Program. We conducted semi-structured interviews in participants’ homes which were audio recorded, transcribed, and analyzed using Dedoose (version 7.0.23). We derived semi-structured interview questions from the Health Belief, Social Cognitive, and Health Action Process Approach framework. We used a structured coding approach using conventional content analysis to derive codes from the text, then applied these codes to each interview and examined the frequency to determine themes. The most frequently referenced theme was barriers to PA, primarily co-morbidities. The primary motivator was social support by a peer or instructor. The preferred PA program components were walking 2-3 days per week with duration varying from 20 minutes to 2 hours. Clients also preferred individualized PA instruction versus a passive strategy such as pamphlets or videotapes. Our findings show that individual-level factors most significantly influence PA participation and should be addressed among Medicaid HCBS clients. We recommend Medicaid HCBS consider a personalized approach of PA implementation with their clients.

